# Class III malocclusion with maxillary deficiency, mandibular prognathism and facial asymmetry

**DOI:** 10.1590/2176-9451.21.5.103-113.bbo

**Published:** 2016

**Authors:** Guilherme de Araújo Almeida

**Affiliations:** 1 Full professor, Universidade Federal de Uberlândia (UFU), School of Dentistry, Uberlândia, Minas Gerais, Brazil.

**Keywords:** Orthodontics, Angle Class III malocclusion, Facial asymmetry.

## Abstract

This article reports the clinical case of a female patient with history of unsuccessful orthodontic treatment. She presented with Class III malocclusion, mandibular and maxillary constriction, anterior crossbite and facial asymmetry resulting from laterognathism triggered by hyperactivity of the condyle revealed by vertical elongation of the right mandibular ramus. Patient's treatment consisted of orthodontic mechanics and two orthognathic surgical interventions with satisfactory and stable outcomes. This case was presented to the Brazilian Board of Orthodontics and Dentofacial Orthopedics (BBO), as part of the requirements for obtaining the BBO Diplomate title.

## RESUMO

Este artigo relata o caso clínico de uma paciente do sexo feminino, com história de insucessos em tratamentos ortodônticos pregressos. Apresentava má oclusão de Classe III, com atresia de ambas as arcadas dentárias, mordida cruzada anterior e assimetria facial, proveniente de laterognatismo desencadeado por uma hiperatividade condilar, manifestada pelo alongamento vertical do ramo mandibular direito. Seu tratamento envolveu a mecanoterapia ortodôntica e duas intervenções cirúrgicas ortognáticas, com resultados considerados satisfatórios e estáveis. Esse caso foi apresentado à Diretoria do Board Brasileiro de Ortodontia e Ortopedia Facial (BBO), como parte dos requisitos para a obtenção do título de Diplomado pelo BBO.

## INTRODUCTION

A female, Caucasian, 20-year and 8-month-old patient presented for initial examination and reported having been subjected to a number of orthodontic and orthopedic treatment modalities since she was seven years old. Her chief complaint was about mandibular laterognathism, and she presented without any family history or previous report of dental and/or facial trauma. Her medical history was nonsignificant or without any association with the issue presented, and there were no correlated symptoms.

## DIAGNOSIS

**Figure 1:**
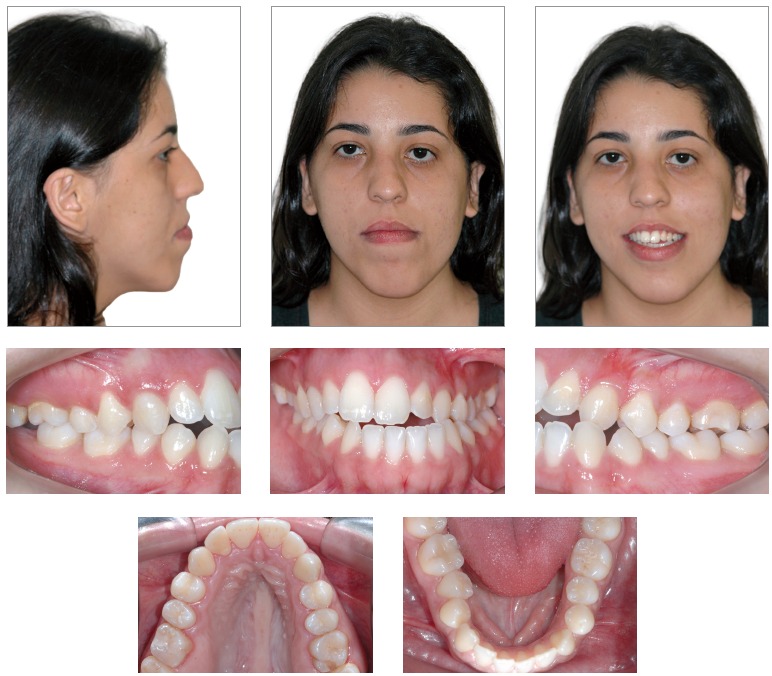
Initial extra and intraoral photographs.

Facial clinical examination revealed the patient presented with facial asymmetry most distinct on the left side, lip incompetence, little zygomatic bone expression, lower sclera exposure and a nearly straight profile, which not only revealed the excessive activity of the mandible and facial vertical components, but also little maxillary expression in its facial scaffold (Fig 1). 

**Figure 2:**
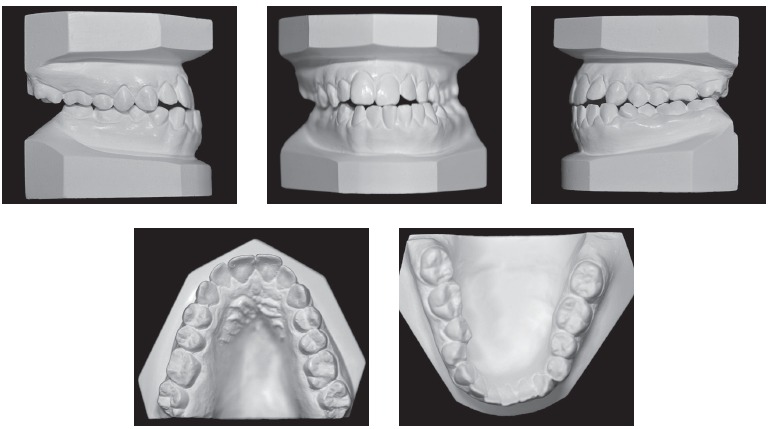
Initial orthodontic study casts.

Dental assessment revealed satisfactory health conditions, without clinical evidence of potential iatrogenesis resulting from the patient's orthodontic/orthopedic history. The patient presented with Angle Class III malocclusion associated with anterior crossbite of central/lateral incisors and canine teeth, all of which belonged to the left upper quadrant; anterior open bite; mandibular midline deviation to the left, which was clinically pronounced by mandibular asymmetry in the opposite direction; mandibular and maxillary transverse constriction and mild crowding in the anterior-mandibular region (Figs 1 and 2). 

**Figure 3:**
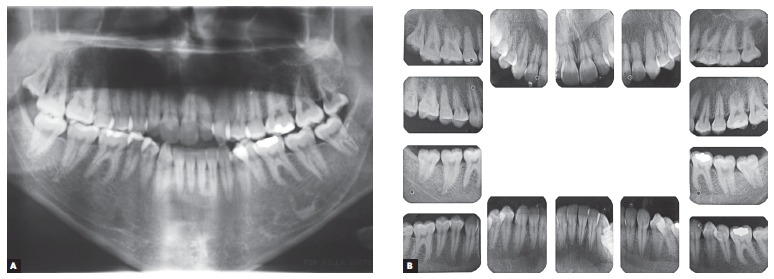
Initial panoramic (A) and periapical (B) photographs.

Supplementary radiographic examination, panoramic and full periapical ones in particular, evinced hemi mandibular elongation on the right side, which explained skeletal asymmetry on the opposite side, associated with rounding of roots, especially of maxillary incisors (Fig 3).

**Figure 4:**
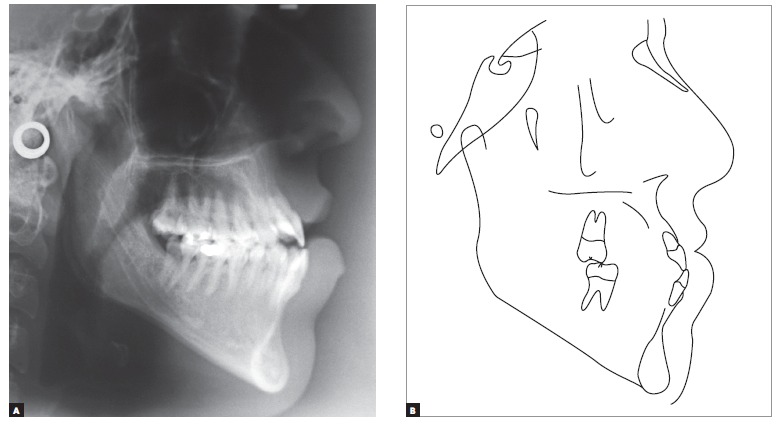
Initial cephalograms in lateral view (A) and cephalometric tracing (B).

Cephalometric analysis revealed Class III skeletal relationship, with proportional maxillary and mandibular protrusion relative to the base of the skull. The SNA value was in disagreement with facial examination suggesting maxillary deficiency, probably due to the length of the base of the skull, which is often short in Class III malocclusion cases, in addition to increased facial height in the anterior-mandibular region (Fig 4, Table 1).

**Table 1:** Initial, preoperative and final cephalometric values.

	Measurements		Normal	Initial	Pre surgical	Final
Skeletal pattern	SNA	(Steiner)	82°	87,0^o^	82,0^o^	89,5^o^
	SNB	(Steiner)	80°	85,0^o^	82,0^o^	84,5^o^
	ANB	(Steiner)	2°	2,0^o^	0,0^o^	5,0^o^
	Angle of convexity	(Downs)	0°	3,0^o^	0,0^o^	3,5^o^
	Y-axis	(Downs)	59°	67,0^o^	64,0^o^	62,0^o^
	Facial angle	(Downs)	87°	90,0^o^	93,0^o^	94,5^o^
	SN-GoGn	(Steiner)	32°	39,0^o^	43,0^o^	33,5^o^
	FMA	(Tweed)	25°	37,0^o^	34,5^o^	28,5^o^
Dental pattern	IMPA	(Tweed)	90°	81,5^o^	84,0^o^	88,0^o^
	1.NA (degrees)	(Steiner)	22°	29,0^o^	29,5^o^	19,5^o^
	1-NA (mm)	(Steiner)	4mm	7,0mm	11,0mm	6,0mm
	1.NB (degrees)	(Steiner)	25°	27,0^o^	32,0^o^	28,0^o^
	1-NB (mm)	(Steiner)	4mm	12,0mm	11,0mm	11,0mm
	- Interincisal angle	(Downs)	130°	121,0^o^	119,0^o^	127,5^o^
	1-APo	(Ricketts)	1mm	9,5mm	11,0mm	5,0mm
Profile	Upper lip - S-line	(Steiner)	0mm	0,0mm	- 1,0mm	1,0mm
	Lower lip - S-line	(Steiner)	0mm	7,0mm	5,0mm	3,0mm

From a functional standpoint, there was neither opening restriction nor signs or symptoms of temporomandibular disorder, except for absence of excursion.

## TREATMENT PLAN

Based on the established diagnosis, treatment objectives were as follows: correction of molar relationship; elimination of dental arch transverse deficiency; correction of anterior open bite; recovery of facial symmetry; tooth alignment and leveling; and establishment of functional excursion. To this end, it was determined that therapeutic intervention should be carried out by means of combining Orthodontics and Orthognathic Surgery. The latter would be performed at two different stages: first with a view to recovering maxillary transverse dimension, and second with a view to recovering both anteroposterior and vertical relationships of the maxillomandibular complex, along with recovery of facial symmetry.

## TREATMENT PROGRESS

Treatment began with direct bonding on maxillary incisors, with central incisors having brackets temporarily placed with greater angulation, so as to provide greater divergence of roots, thus assisting the job of the professional responsible for carrying out the surgically-assisted maxillary expansion. Once divergence of roots between the aforementioned teeth was achieved, a Haas appliance was placed, banded on maxillary first molars and first premolars, with an activation of 2/4 of a turn/day, with a total of four complete turns until overcorrection was achieved. Overcorrection was checked by means of previous upper arch impression associated with a previous lower arch dental cast. By the end of the active period, retention was performed with the Haas appliance which was used for six months. Once this treatment phase had been concluded, a Porter arch (W-shaped) was placed as a supplementary stabilization device during the early stages of tooth alignment and leveling, both carried out by means of a fixed appliance with the Straight-wire technique and slot 0.022 x 0.028-in.

In the case reported herein, tooth alignment and leveling firstly aimed at eliminating dental compensation, thus increasing lower arch circumference and uprighting maxillary incisors. In order to check for success at this phase, consecutive impressions of both arches were taken, in addition to articulated casts, so as to simulate surgical movement of the maxillomandibular complex.

**Figure 5:**
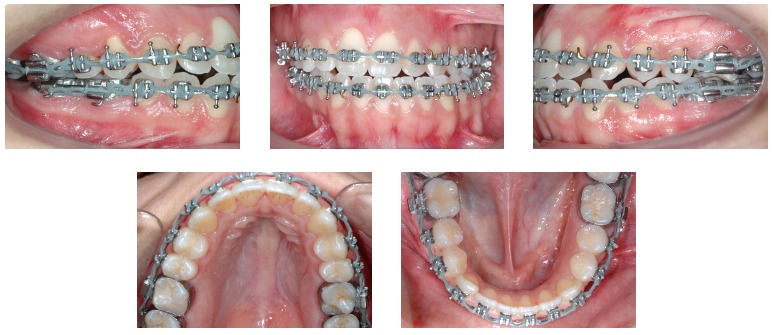
Intraoral photographs before surgery.

**Figure 6:**
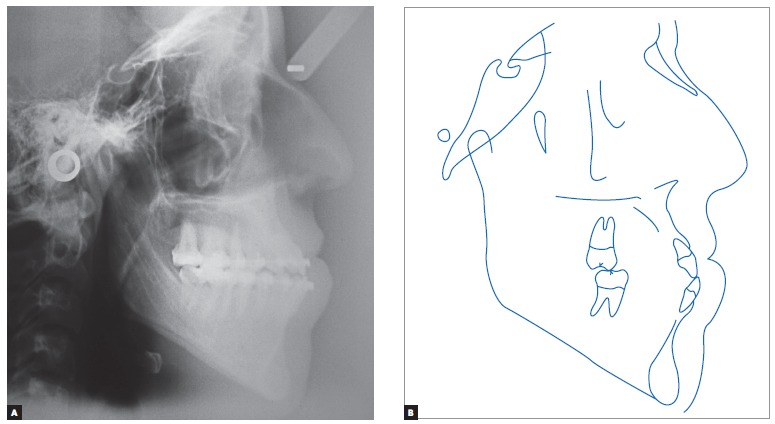
Initial cephalograms in lateral view (A) and cephalometric tracing (B) before surgery.

**Figure 7:**
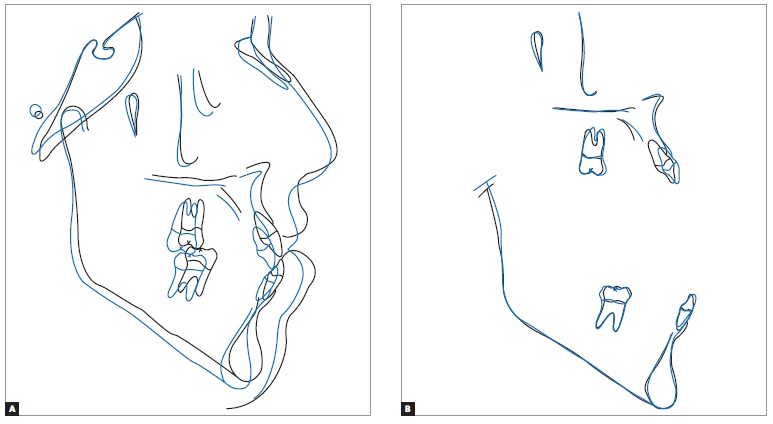
Total (A) and partial (B) superimposition of initial (black) and pre-surgical (blue) cephalometric tracings.

Once dental decompensation of both arches had been achieved (Figs 5, 6 and 7, Table 1), the patient was subjected to orthognathic surgery which consisted of maxillary advancement and impaction, especially on the right side, decrease of mandibular prognathism and correction of asymmetry, in addition to advancement and decrease of chin height. After surgical recovery completion, the patient was subjected to mechanics with Class III elastics followed by intercuspation, so as to achieve satisfactory interocclusal relationship and masticatory function during mandibular excursion.

## RESULTS

Esthetic outcomes were considered satisfactory. In frontal view, asymmetry was corrected; whereas in lateral view, pleasant profile smoothing was achieved (Fig 9).

**Figure 8:**
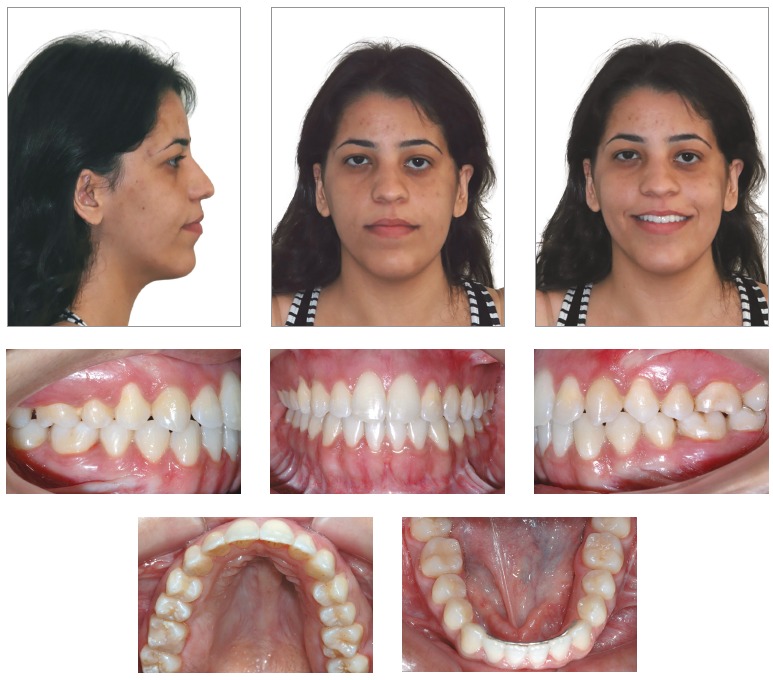
Final extra and intraoral photographs.

**Figure 9:**
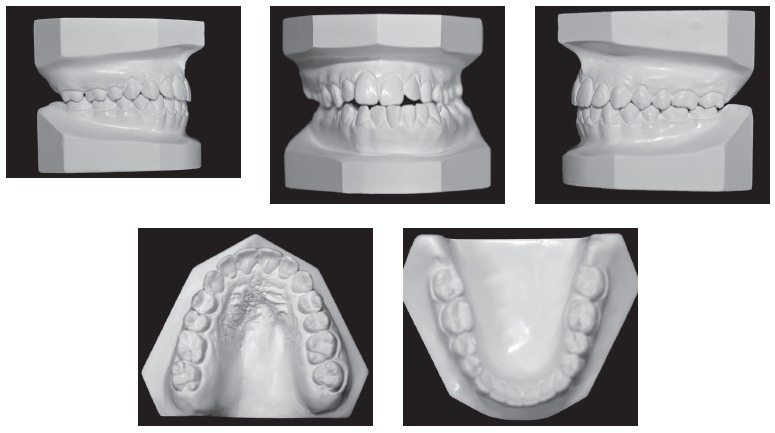
Final orthodontic study casts.

Both arches had their transverse dimensions restored. Maxillary incisors had buccal tipping and protrusion decreased, whereas mandibular incisors position on the long axis was slightly decreased, with protrusion remaining unchanged (Figs 8, 9 and 11, Table 1). Such dental positioning provided favorable conditions to achieve functional excursion, which is in accordance with the initial treatment goals.

**Figure 10:**
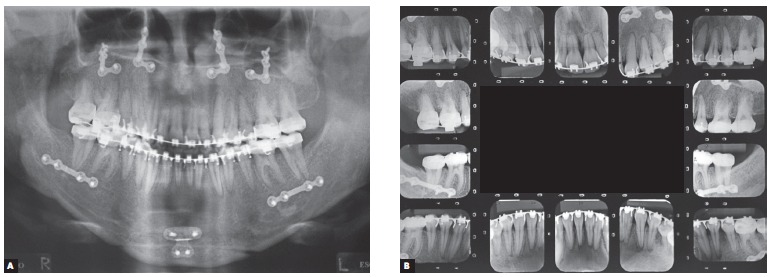
Final panoramic (A) and periapical (B) photographs.

**Figure 11:**
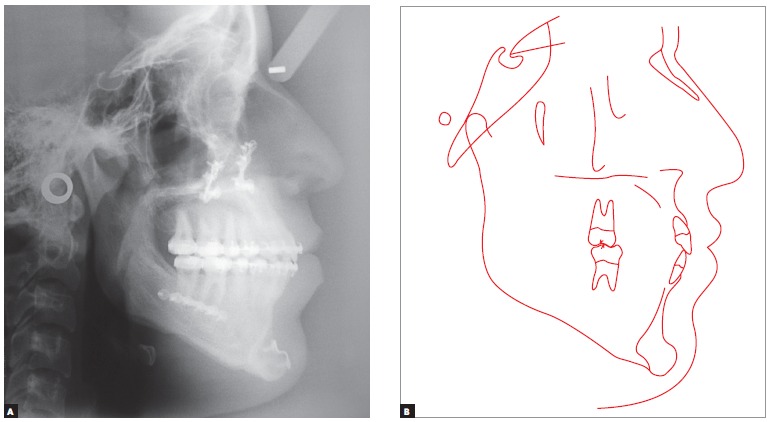
Final cephalograms in lateral view (A) and cephalometric tracing (B).

Although maxillary incisors rounding would allow us to expect a greater decrease in the root length of teeth, that was not consistently revealed by final radiographic examination, as shown in Figure 10.

**Figure 12:**
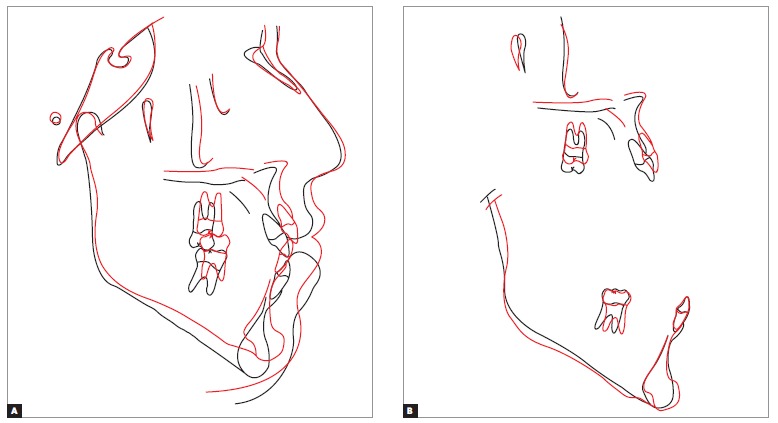
Total (A) and partial (B) superimposition of initial (black) and final (red) cephalometric tracings.

From a skeletal standpoint, the maxilla was more protruded in relation to the base of the skull, as a result of surgical advancement. Although the mandible decreased in size, protrusion remained unchanged, based on the SNB value, due to counterclockwise rotation to which it was subjected. Facial horizontal planes (SN-Go-Gn, Frankfort mandibular plane angle - FMA) were significantly decreased (Figs 11 and 12, Table 1). 

For retention purposes, a 3x3 appliance was placed in the lower arch while a Hawley retainer was placed in the upper arch.

## FINAL CONSIDERATIONS

Mandibular skeletal asymmetry, occasionally caused by hyperactivity in one of the condyles, is of unknown origin, although it might be associated with trauma, inflammation, hypervascularization on the head of the affected jaw, or genetic/hormonal influence.^1^ ^,^ ^2^ ^,^ ^3^ It can be classified as acquired or in development. The former presents with anatomical-physiological changes in the temporomandibular joint (TMJ) associated with painful symptoms. On the contrary, in the latter, the joint condition remains preserved, without any evident symptom.^3^

Whenever present, hemi mandibular hyperactivity can be characterized by generalized increase in volume (hyperplasia), ramus elongation in isolation or by a combination of both.^2^ ^,^ ^3^

In this specific case, as revealed by radiographic images (Figs 3 and 4) and absence of pain or anatomical-functional limitations of the TMJs, hyperactivity of the condyle is in development and characterized by hemi mandibular elongation on the right side. Should this pathology be present, the orthodontist, whenever identifying the increasing manifestation of mandibular asymmetry, must have treatment discontinued and request diagnostic examination, such as bone scintigraphy, capable of evincing the presence of osteoblastic activity in the TMJ. In the event of positive examination results, it is recommended that orthodontic intervention be resumed only after future examination evince that hyperplastic pathological activity has ceased. Should it not occur after a significant period of time, the feasibility of performing surgical access to the affected condyle can be considered, in accordance with the orthognathic surgeon and once craniofacial growth has ceased. In the clinical case reported herein, after a follow-up period and according to previous records, facial asymmetry stability was evinced, which induced orthodontic/orthognathic surgical treatment to be carried out.

**Figure 13:**
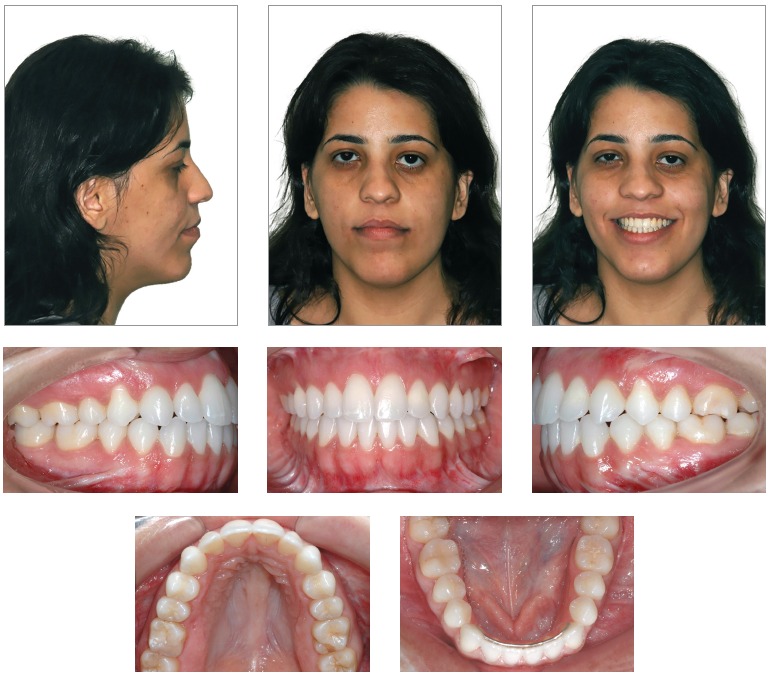
Extra and intraoral photographs 6 years after treatment.

Finally, although there is a potential for other manifestations of hyperactivity of the condyle, the reported case proved clinically stable by the end of the follow-up period six years after treatment finishing, as shown in Figure 13.
